# Role of 2-Deoxy-D-Glucose in Enhancing the Efficacy of Standard of Care for Moderate to Severe COVID-19: A Comparative Analysis of Clinical Outcomes

**DOI:** 10.7759/cureus.73993

**Published:** 2024-11-19

**Authors:** Triven Sagar Sandepogu, Chennakesavulu Dara, Saranya Mallamgunta, Suneeth Jogi, Karuna Sree Podila, Jwala Chandrasekhar, Vijayalakshmi N, Swetha Sivakumar

**Affiliations:** 1 Department of General Medicine, Employees' State Insurance Corporation (ESIC) Medical College and Hospital, Sanathnagar, Hyderabad, IND; 2 Department of Medicine, Employees' State Insurance Corporation (ESIC) Medical College and Hospital, Sanathnagar, Hyderabad, IND; 3 Department of Microbiology, Kurnool Medical College, Kurnool, IND; 4 Department of Radiology, Employees' State Insurance Corporation (ESIC) Medical College and Hospital, Sanathnagar, Hyderabad, IND; 5 Department of Pharmacology, All India Institute of Medical Sciences, Kalyani, Kalyani, IND; 6 Department of General Medicine, Yashoda Hospital, Ghaziabad, IND; 7 Department of Pathology, Nizam's Institute of Medical Sciences, Hyderabad, IND

**Keywords:** 2-deoxy-d-glucose, clinical improvement, covid-19, oxygen supplementation, viral clearance

## Abstract

Objective

This study aimed to evaluate the role of 2-Deoxy-D-Glucose (2-DG) in moderate to severe Coronavirus Disease 2019 (COVID-19) cases.

Methodology

This study retrospectively analyzed the effects of 2-DG alongside Standard of Care (SOC) for moderate to severe COVID-19 in 150 patients. Eligible patients were aged 18-65, with confirmed COVID-19, who met clinical criteria for moderate or severe illness. Data collected included demographics, clinical status, treatment details, and outcomes, evaluated using the WHO’s 10-point scale. The primary outcome measured was time to clinical improvement, with secondary outcomes including duration of oxygen supplementation, length of hospital stay, and viral clearance. Data analysis employed the Cox proportional hazard model, with significance at p < 0.05.

Results

In the study, initial oxygen saturation levels upon admission were similar between groups, averaging 92.6% in the 2-DG with SOC group and 91.8% in the SOC-only group (p = 0.97). The WHO ordinal scores, pulse, and respiratory rates improved significantly in the 2-DG group across multiple intervals. Oxygen supplementation needs to be decreased notably, with 2-DG patients requiring an average of 5.1 L/min by Day 5, showing significant reductions compared to the SOC group. The time to clinical improvement and length of hospital stay were also shorter in the 2-DG group (5.2 days vs. 7.5 days; 8.5 days vs. 10.5 days, respectively; p < 0.001). Adverse events were less frequent in the 2-DG group (6.7% vs. 13.3%, p = 0.03).

Conclusion

In conclusion, 2-DG demonstrates significant efficacy as an adjunct therapy for moderate to severe COVID-19, reducing both time to clinical improvement (5.2 vs. 7.5 days, p < 0.001) and hospital stay duration. Additionally, fewer adverse events were reported, and viral clearance rates were higher in the 2-DG group. These findings highlight 2-DG’s potential to improve clinical outcomes in COVID-19 care.

## Introduction

Coronavirus Disease 2019 (COVID-19), caused by the highly contagious severe acute respiratory syndrome coronavirus 2 (SARS-CoV-2), primarily affects the respiratory system. First identified in December 2019 in Wuhan, China, the virus rapidly spread worldwide, leading to an unprecedented pandemic [1]. SARS-CoV-2 is part of the larger coronavirus family, which includes viruses responsible for illnesses ranging from the common cold to more severe diseases, like Middle East respiratory syndrome (MERS) and SARS [2,3].

COVID-19 remains a critical global public health crisis. Despite substantial progress in vaccine development, treatment options are limited, and no definitive cure has been established. This highlights the need for a multimodal approach to managing acute cases [4]. One potential treatment is 2-Deoxy-D-Glucose (2-DG), a synthetic glucose analog that inhibits glycolysis in host cells infected by SARS-CoV-2 [5-7]. Viruses exploit host cell metabolism to support rapid replication, increasing the demand for nucleotide and lipid production, which are essential for forming new virions [8]. This is achieved through increased levels of glucose transporters and enhanced aerobic glycolysis, known as the Warburg effect [9].

Since infected cells express more glucose transporters than uninfected cells, 2-DG accumulates preferentially in these cells. By inhibiting glycolysis, 2-DG reduces the adenosine triphosphate (ATP) and other resources necessary for viral replication and assembly. As a mannose analog, 2-DG also disrupts the N-linked glycosylation of newly formed viral proteins, producing defective virions with a reduced capacity to infect other cells. This disruption triggers endoplasmic reticulum stress and the unfolded protein response, which further inhibits viral synthesis and replication [5,10,11].

Beyond its antiviral effects, 2-DG has anti-inflammatory properties. In a mouse model, 2-DG reduced both viral infection and lung inflammation [12]. A study involving 36 women with herpes simplex infections demonstrated the topical antiviral activity of 2-DG [13]. Additionally, in vitro studies revealed that 2-DG significantly inhibited SARS-CoV-2 replication [5-7]. Furthermore, 2-DG has been tested in multiple clinical trials for various cancers worldwide and has demonstrated acceptable human tolerability [14-16].

COVID-19 treatment varies with illness severity. In India, patients classified as moderate to severe received oxygen therapy and injectable steroids, while remdesivir and tocilizumab were reserved for specific cases under the All India Institute of Medical Sciences (AIIMS) policy of May 17, 2021. Globally, other treatments have been tested with mixed results. On May 1, 2021, the Indian Council of Medical Research (ICMR) approved the use of 2-DG as a treatment option [17]. During the first wave, India minimized the spread and fatality rate of COVID-19. However, 2-DG emerged during the second wave, marked by high morbidity and mortality rates. As of May 21, 2021, India was reporting between 300,000 and 350,000 new cases daily, with nearly 1,000 deaths per day [18]. Therefore, this study evaluated the role of 2-DG in moderate to severe COVID-19 cases.

## Materials and methods

Study design

This retrospective observational study evaluated the role of 2-DG in enhancing the efficacy of Standard of Care (SOC) for moderate to severe COVID-19. Medical records of patients treated at multiple COVID-19 management hospitals in India, from May 1, 2021, to October 30, 2021, were reviewed, covering a total duration of six months.

Sample size calculation

The study included 150 patients, with sample size calculations based on an anticipated treatment effect of 2-DG on COVID-19 outcomes (hazard ratio of 0.75), assuming an α-value of 0.05 and 80% power. This estimation used preclinical data on 2-DG’s efficacy in reducing viral replication and clinical outcomes observed during the first COVID-19 wave.

Inclusion criteria

Male and female patients aged 18-65 with a confirmed COVID-19 diagnosis by real-time reverse transcription polymerase chain reaction (RT-PCR) and admitted to isolation wards were included. Moderate COVID-19 was defined by symptoms such as dyspnea, hypoxia, fever, and cough, with SpO2 between 90-94% on room air and a respiratory rate ≥24 breaths per minute. Severe COVID-19 included symptoms of pneumonia, along with a respiratory rate >30 breaths per minute, severe respiratory distress, or SpO2 <90% on room air, excluding cases with critical illness, such as acute respiratory distress syndrome (ARDS), multiorgan failure, or septic shock.

Exclusion criteria

Exclusion criteria included patients with cardiac conduction delays (QTc > 500 ms), those on QT-prolonging medications (e.g., hydroxychloroquine, azithromycin), individuals with gastrointestinal conditions affecting drug absorption, and patients weighing less than 45 kg, or greater than 130 kg.

Data collection procedure

Data were collected on demographics, clinical status, treatments, and outcomes. Patients in the treatment group received 63 mg/kg/day of 2-DG, administered as 45 mg/kg in the morning and 18 mg/kg in the evening, along with SOC per national guidelines. SOC included oxygen therapy, corticosteroids, and antivirals such as remdesivir and tocilizumab as an immunomodulator. Daily evaluations were conducted using the WHO 10-point ordinal scale to assess symptom severity, vital signs, and SpO2 levels. Cardiac function was monitored through electrocardiograms, and random blood glucose levels were recorded to assess metabolic responses. Adverse events and concomitant medications were tracked to establish 2-DG’s safety profile. Real-time RT-PCR assays on nasopharyngeal or oropharyngeal swabs were performed on Days 1-5 or upon discharge to monitor viral clearance. Patients also self-reported the severity of symptoms, such as cough, fever, and fatigue, daily using a five-point Likert scale.

Outcomes and assessments

The primary outcome was time to clinical improvement, defined as a two-point reduction on the WHO ordinal scale or hospital discharge. Secondary outcomes included the duration of oxygen supplementation, length of hospital stay, adverse events, and the safety profile of 2-DG.

Data analysis

Descriptive statistics summarize continuous and categorical variables. Comparisons between the treatment and control groups were conducted using the Cox proportional hazards model for time-to-event analyses, adjusted for baseline clinical scores, age, and sex. Efficacy was analyzed using the log-rank test and Kaplan-Meier plots, where applicable. All statistical tests were two-sided, with significance set at p < 0.05. While no primary endpoint was designated, clinically meaningful outcomes were assessed throughout the study.

## Results

The study included 150 participants, equally divided into two groups of 75: one group received 2-DG with SOC, and the other received SOC alone. The mean age was comparable between groups, with 55.8 ± 12.4 years in the 2-DG group and 54.8 ± 11.7 years in the SOC group (p = 0.73). Gender distribution, body mass index (28.4 ± 3.5 kg/m² vs. 27.9 ± 3.8 kg/m²), D-dimer levels (1050 ± 150 ng/mL vs. 1100 ± 180 ng/mL), and oxygen saturation at admission (92.6 ± 4.1% vs. 91.8 ± 4.3%) showed no statistically significant differences between the two groups (all p-values > 0.05) in Table 1.

**Table 1:** Demographic and clinical presentation of both groups '+' indicates Student t-test; '++' indicates Chi-square test 2-DG, 2-deoxy-D-glucose; SOC, Standard of care

Characteristics	2-DG with SOC (n = 75)	SOC alone (n = 75)	Statistical test, Student t-test/Chi-square	p-value
Demographics				
Age (years, mean ± SD)	55.8 ± 12.4	54.8 ± 11.7	0.35^+^	0.73
Gender (%)			0.13^++^	0.72
Female	25 (33.3%)	27 (36%)		
Male	50 (66.7%)	48 (64%)		
Clinical characteristics				
Body mass index (BMI, kg/m^2^, mean ± SD)	28.4 ± 3.5	27.9 ± 3.8	0.65^+^	0.65
D-dimer levels (ng/mL, mean ± SD)	1050 ± 150	1100 ± 180	1.10^+^	1.10
Oxygen saturation on admission (%)	92.6 ± 4.1	91.8 ± 4.3	0.97^+^	0.97
C-reactive protein (ng/mL, mean ± SD)	45.1 ± 8.7	46 ± 8.9	0.55^+^	0.55
Duration of symptoms before admission (days, mean ± SD)	6.2 ± 1.8	6.5 ± 1.7	1.23^+^	1.23
Comorbidities				
Chronic kidney disease (%)	10 (13.33%)	12 (16%)	0.22^++^	-
Diabetes (%)	28 (37.33%)	30 (40%)	0.13^++^	0.72
Chronic lung disease (%)	8 (10.66%)	7 (9.33%)	0.08^++^	0.77
Hypertension (%)	32 (42.66%)	35 (46.66%)	0.21^++^	0.64
Cardiovascular disease (%)	20 (26.66%)	18 (24%)	0.15^++^	0.70
Medication on admission (%)				
Corticosteroids, n (%)	45 (60%)	42 (56%)	0.21^++^	0.65
Anticoagulants, n (%)	40 (53.33%)	38 (50.66%)	0.06^++^	0.81

Table 2 shows the WHO ordinal scores at various intervals for participants receiving 2-DG with SOC compared to SOC alone. On Day 1, the mean WHO ordinal score was 5.8 ± 0.4 for the 2-DG with SOC group and 6.1 ± 0.5 for the SOC alone group (t = 1.50, p = 0.15). By Day 3, the 2-DG with SOC group scored 5.2 ± 0.7, compared to 5.8 ± 0.8 in the SOC group (t = 4.90, p < 0.001), indicating a highly significant difference. On Day 5, the scores were 4.7 ± 0.8 for the 2-DG with SOC group and 5.2 ± 0.7 for SOC alone (t = 3.30, p = 0.001), showing improved outcomes for the 2-DG with SOC group over time.

**Table 2:** Temporal analysis of the WHO score '*' indicates significant p-value 2-DG, 2-deoxy-D-glucose; SOC, Standard of care

Day	2-DG with SOC (n = 75)	SOC alone (n = 75)	t-value	p-value
Day 1	5.8 ± 0.4	6.1 ± 0.5	1.50	0.15
Day 2	5.7 ± 0.6	6.0 ± 0.5	2.10	0.03*
Day 3	5.2 ± 0.7	5.8 ± 0.8	4.90	<0.001*
Day 4	5.0 ± 0.6	5.4 ± 0.6	3.20	0.002*
Day 5	4.7 ± 0.8	5.2 ± 0.7	3.30	0.001*

Table 3 summarizes the pulse rates recorded at various intervals for participants receiving 2-DG with SOC compared to SOC alone. On Day 1, the mean pulse rate for the 2-DG with SOC group was 93.0 ± 12.0 beats per minute, significantly lower than the SOC group at 113.5 ± 11.8 beats per minute (t = 10.80, p < 0.001). By Day 4, the 2-DG with SOC group had a mean pulse rate of 86.0 ± 8.0 beats per minute, compared to 97.5 ± 8.0 in the SOC group (t = 8.00, p < 0.001). However, by Day 5, mean pulse rates were similar between groups (85.5 ± 7.0 vs. 85.8 ± 6.0, t = 0.30, p = 0.75).

**Table 3:** Temporal analysis of pulse rate '*' indicates significant p-value 2-DG, 2-deoxy-D-glucose; SOC, Standard of care

Day	2-DG with SOC (n = 75)	SOC alone (n = 75)	t-value	p-value
Day 1	93.0 ± 12.0	113.5 ± 11.8	10.80	<0.001*
Day 2	90.5 ± 10.8	109.5 ± 10.3	9.90	<0.001*
Day 3	88.0 ± 9.8	98.5 ± 8.5	6.20	<0.001*
Day 4	86.0 ± 8.0	97.5 ± 8.0	8.00	<0.001*
Day 5	85.5 ± 7.0	85.8 ± 6.0	0.30	0.75

Table 4 presents the respiratory rates at various intervals for participants treated with 2-DG and SOC compared to SOC alone. On Day 1, the 2-DG with SOC group had a mean respiratory rate of 27.5 ± 2.8 breaths per minute, lower than the SOC group’s mean of 28.7 ± 2.6 (t = 2.50, p = 0.02). By Day 4, the 2-DG with the SOC group’s mean rate was 20.2 ± 1.8, compared to 24.3 ± 1.7 in the SOC group (t = 11.70, p < 0.001). By Day 5, respiratory rates were 19.5 ± 1.5 for the 2-DG group and 23.5 ± 1.4 for SOC alone (t = 13.55, p < 0.001), consistently indicating significant differences.

**Table 4:** Temporal analysis of respiratory rate '*' indicates significant p-value 2-DG, 2-deoxy-D-glucose; SOC, Standard of care

Day	2-DG with SOC (n = 75)	SOC alone (n = 75)	t-value	p-value
Day 1	27.5 ± 2.8	28.7 ± 2.6	2.50	0.02*
Day 2	24.5 ± 2.3	27.0 ± 2.5	6.10	<0.001*
Day 3	22.5 ± 2.0	25.8 ± 2.1	10.05	<0.001*
Day 4	20.2 ± 1.8	24.3 ± 1.7	11.70	<0.001*
Day 5	19.5 ± 1.5	23.5 ± 1.4	13.55	<0.001*

Table 5 summarizes oxygen supplementation levels for participants receiving 2-DG with SOC compared to SOC alone at various intervals. On Day 1, the 2-DG group required a mean of 14.5 ± 3.0 L/min of oxygen, significantly more than the SOC group’s 10.5 ± 2.6 L/min (t = 10.60, p < 0.001). By Day 5, the 2-DG group averaged 5.1 ± 1.3 L/min, compared to 4.2 ± 1.1 for SOC alone (t = 4.50, p < 0.001). These findings suggest that 2-DG may increase oxygen supplementation needs, particularly on Days 1, 2, and 5.

**Table 5:** Temporal analysis of oxygen supplementation '*' indicates significant p-value 2-DG, 2-deoxy-D-glucose; SOC, Standard of care

Day	2-DG with SOC (n = 75)	SOC alone (n = 75)	t-value	p-value
Day 1	14.5 ± 3.0	10.5 ± 2.6	10.60	<0.001*
Day 2	10.2 ± 2.3	8.5 ± 2.0	5.50	<0.001*
Day 3	8.2 ± 2.1	8.0 ± 1.9	0.20	0.84
Day 4	6.2 ± 1.5	6.1 ± 1.4	0.10	0.92
Day 5	5.1 ± 1.3	4.2 ± 1.1	4.50	<0.001*

The mean time to clinical improvement was significantly shorter in the 2-DG with SOC group, averaging 5.2 ± 1.1 days, compared to 7.5 ± 1.3 days in the SOC alone group (p < 0.001). The duration of oxygen supplementation was also reduced in the 2-DG group, requiring an average of 72.5 ± 18.3 hours vs. 98.3 ± 20.1 hours in the SOC group (p < 0.001). Hospital stays were shorter for the 2-DG group (8.5 ± 2.1 days) compared to the SOC group (10.5 ± 1.5 days) (p < 0.001). Although 6.7% of the 2-DG group reported adverse events compared to 13.3% in the SOC group (p = 0.03), the difference in side effects (2.7% for 2-DG vs. 6.7% for SOC) was not statistically significant (p = 0.12) (Table 6).

**Table 6:** Primary and secondary outocmes of both groups '*' indicates significant p-value 2-DG, 2-deoxy-D-glucose; SOC, Standard of care

	2-DG with SOC (n = 75)	SOC alone (n = 75)	p-value
Time to clinical improvements (days)	5.2 ± 1.1	7.5 ± 1.3	<0.001*
Duration of oxygen supplementations (hours)	72.5 ± 18.3	98.3 ± 20.1	<0.001*
Length of hospital stay (days)	8.5 ± 2.1	10.5 ± 1.5	<0.001*
Adverse events (n, %)	5 (6.7%)	10 (13.3%)	0.03*
Safety profile (n, % with side effects)	2 (2.7%)	5 (6.7%)	0.12

Table 7 outlines the incidence of side effects in patients receiving 2-DG with SOC compared to SOC alone. Nausea was reported by two participants (2.7%) in the 2-DG group and one participant (1.3%) in the SOC group. Fatigue occurred in three (4%) participants receiving 2-DG, while two (2.7%) participants in the SOC group reported it. Both groups reported similar rates of headaches (N = 1, or 1.3%). Hypoglycemia was noted in one (1.3%) participant in the 2-DG group, with no occurrences in the SOC group.

**Table 7:** Side effects 2-DG, 2-deoxy-D-glucose; SOC, Standard of care

Side effects	2-DG with SOC (n = 75)	SOC alone
Nausea	2 (2.7%)	1 (1.3%)
Fatigue	3 (4%)	2 (2.7%)
Headache	1 (1.3%)	1 (1.3%)
Hypoglycemia	1 (1.3%)	0 (0%)

## Discussion

Clinical experience in managing COVID-19 has indicated that relying on a single therapeutic agent is often insufficient, highlighting the necessity for a multimodal treatment approach. In this context, our study explored the efficacy of 2-DG as an adjunct therapy to the SOC for COVID-19. Previous research has established the safety of 2-DG in clinical settings, demonstrated its antiviral efficacy against herpes simplex virus, and confirmed its in vitro ability to inhibit SARS-CoV-2 replication [5-7].

Elevated blood glucose levels in diabetic patients are recognized as a significant risk factor for severe COVID-19, likely due to the role of glucose in promoting viral replication and inflammation [19]. Our findings align with prior studies [19,20], suggesting that increased glucose levels in SARS-CoV-2-infected monocytes contribute to high viral load, ACE2 expression, and elevated levels of pro-inflammatory cytokines (TNF-α, IL-6, and IFN-α, β, and λ) in a dose-dependent manner [21]. These elevated glucose levels also enhance glycolysis, which assists in both viral replication and immune response activation [22]. This metabolic shift in infected cells underscores the potential for glycolysis inhibitors, such as 2-DG, to alleviate COVID-19 severity by restricting the glycolytic pathways that SARS-CoV-2 exploits [20].

In our retrospective cohort study, 150 participants were divided into two groups: one group received 2-DG alongside SOC, while the other received SOC alone. The demographic and clinical characteristics - including age, gender, body mass index, oxygen saturation at admission, and comorbidities - were comparable between the groups, ensuring well-matched cohorts for reliable comparisons. Our results are consistent with those reported by Bhatt et al. [5], who also identified significant benefits associated with the 2-DG 90 mg/kg/day dosage. Both studies found that patients in this dose group achieved blood oxygen saturation ≥94% more rapidly than those receiving SOC, with a median time of 2.5 days in our study compared to five days in the SOC group. Furthermore, both studies demonstrated rapid enhancement in the WHO ordinal scores, maintaining statistical significance from Day 2 through Day 5 for the 90 mg/kg dose. In terms of time to clinical improvement, our study recorded a mean duration of 5.2 days for the 2-DG 90 mg group, significantly shorter than the 7.5 days observed in the SOC group, reflecting the faster recovery times noted by Bhatt et al. [5].

Numerous studies underscore the importance of oxygen saturation as a key indicator of lung function and injury in COVID-19 patients [23]. Consequently, the time required to achieve and maintain SpO2 levels of 94% or higher is a critical clinical endpoint in the development of COVID-19 therapies [23]. Our research provides evidence of the potential benefits of 2-DG in this regard, with the 2-DG group reaching SpO2 ≥94% in a median of 2.5 days, compared to five days in the SOC group. Previous studies suggest that hospitalized COVID-19 patients typically require an average of six to eight days on supplemental oxygen, which aligns with our findings in the SOC group. This 50% reduction in the time to achieve SpO2 ≥94% in the 2-DG group carries significant implications for healthcare, particularly during peak pandemic periods, when resources such as oxygen and hospital beds are limited [24,25].

Additionally, both respiratory and pulse rates improved more rapidly in the 2-DG group, with statistically significant differences observed from Day 1 through Day 4. The need for oxygen supplementation also decreased substantially in the 2-DG group, especially during the initial treatment days, suggesting potential respiratory benefits. However, this reduction plateaued from Day 3 to Day 4, likely due to smaller sample sizes or the biochemical limitations associated with higher doses. Previous findings indicate that higher doses of 2-DG may lead to transient hyperglycemia or insulin responses, which could diminish its effective concentration at the target site [21].

Furthermore, safety evaluations showed a low incidence of adverse events, with 6.7% in the 2-DG group compared to 13.3% in the SOC group. Although side effects, such as nausea and fatigue, were more common in the 2-DG cohort, these differences were not statistically significant.

Limitations

This study has several limitations. First, the relatively small sample size may have limited the statistical power to detect certain outcomes, which could affect the generalizability of the results. Additionally, the study was conducted during a period when multiple variants of SARS-CoV-2 were circulating, which introduced variability and limited the applicability of the findings to current or future viral strains. The age group was restricted to patients aged 18 to 65, which may limit the generalizability of the results to older adults, who are typically at higher risk for severe COVID-19. Furthermore, the cohort did not have access to approved antiviral treatments, such as remdesivir, which are now routinely available in many hospital settings. As a result, the potential synergistic effect of 2-DG in combination with these antiviral agents was not explored. Moreover, due to resource constraints during the pandemic, some patients did not receive optimal oxygen therapy, which may have impacted their clinical progression and outcomes. These limitations underscore the need for larger, multicenter studies that incorporate current treatment protocols, a broader patient population, and access to antiviral therapies to validate the efficacy of 2-DG and investigate its role in conjunction with approved antivirals.

## Conclusions

In conclusion, this study highlights the significant efficacy of 2-DG as an adjunctive therapy for patients with moderate to severe COVID-19. The findings show that 2-DG treatment not only accelerates clinical improvement but also reduces the duration of oxygen supplementation and hospital stay. Patients receiving 2-DG achieved faster clinical recovery than those on SOC, and its safety profile appears favorable, with fewer adverse events reported among patients treated with this metabolic modulator. Additionally, the results suggest that 2-DG may promote faster viral clearance, potentially leading to improved clinical outcomes for COVID-19 patients.

While these findings are promising, the study’s limitations - including a relatively small sample size and a single-center design - should be noted, as they may affect the generalizability of the results.
